# Oxygen Overshoot: A Case Report Navigating the Perils of Unsupervised Supplementation in Chronic Obstructive Pulmonary Disease (COPD)

**DOI:** 10.7759/cureus.50274

**Published:** 2023-12-10

**Authors:** Ekrem Yetiskul, Seth Lisle, Taqi A Rizvi, Shahkar Khan, Gregory A Maniatis

**Affiliations:** 1 Internal Medicine, Staten Island University Hospital-Northwell Health, New York, USA; 2 Internal Medicine, Touro College of Osteopathic Medicine, New York, USA; 3 Cardiology, Staten Island University Hospital-Northwell Health, New York, USA

**Keywords:** haldane effect, pulmonary physiology, copd (chronic obstructive pulmonary disease), oxygen therapy, pulmonary critical care

## Abstract

Supplemental oxygen administration is a delicate balance in managing chronic obstructive pulmonary disease (COPD), where the risk of exacerbating hypercapnia must be carefully considered. This case report describes a 69-year-old male with COPD who, after self-medicating with commercially available portable oxygen bottles, experienced hypercapnic respiratory failure and severe respiratory acidosis, leading to intensive care unit (ICU) admission and non-invasive ventilation. The patient's unsupervised use of commercially available portable oxygen bottles emphasizes the risks associated with unregulated supplemental oxygen in COPD. This case highlights the critical importance of cautious oxygen supplementation in COPD, urging high-risk patients to seek medical guidance, even with over-the-counter products. This case emphasizes the need for expert medical opinion to ensure safe oxygen use in vulnerable populations.

## Introduction

Providing supplemental oxygen can be a careful balance between treating chronic hypoxemia and exacerbating hypercapnia in patients with chronic obstructive pulmonary disease (COPD) [1,2]. Uncontrolled administration of oxygen can induce hypercapnia in patients who are at high risk [1]. Careful consideration should be taken when companies directly advertise supplemental oxygen as a commercially available respiratory therapy. 

We present a case of a 69-year-old male with COPD presenting to the intensive care unit (ICU) after self-medicating with commercially available portable oxygen bottles, resulting in subsequent hypercapnic respiratory failure and severe respiratory acidosis requiring non-invasive ventilation.

## Case presentation

**Figure 1:**
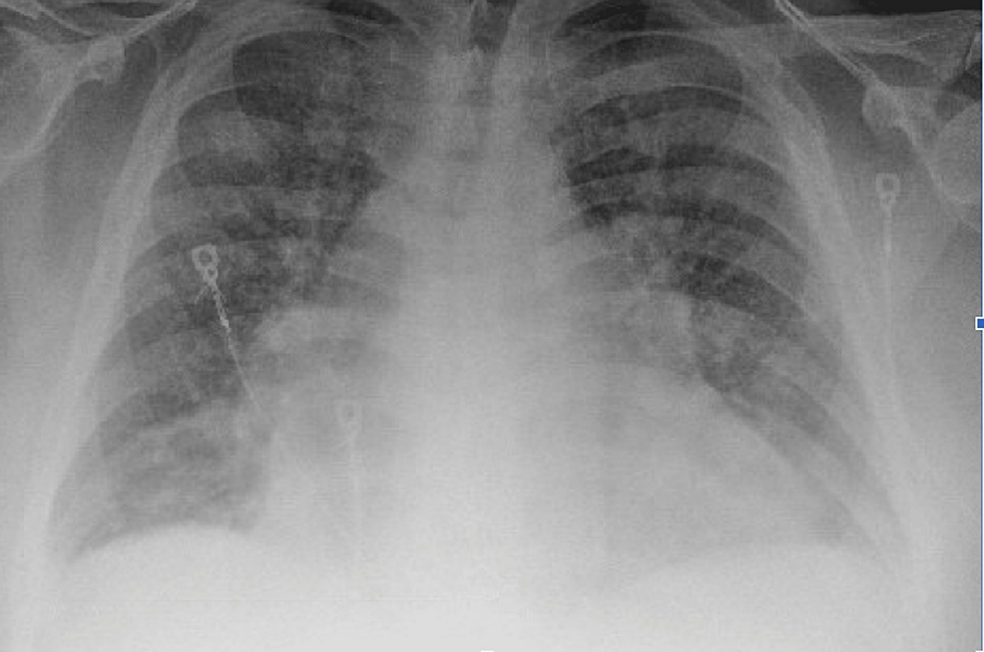
Initial chest x-ray demonstrating an enlarged heart and bilateral opacifications consistent with congestive heart failure

**Figure 2:**
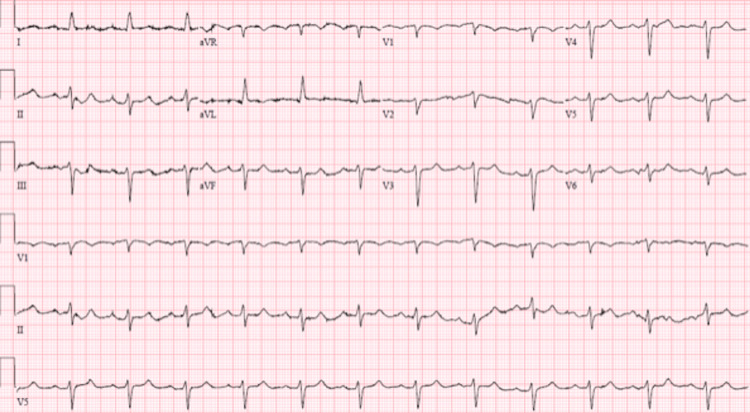
Electrocardiogram performed on admission showing normal sinus rhythm and left axis deviation

We present a 69-year-old male with a past medical history of chronic heart failure with reduced ejection fraction, atrial fibrillation, gout, morbid obesity with a body mass index (BMI) of 45.3, and COPD without prescribed home oxygen supplementation. The patient initially presented to the emergency department with a two-week history of increasing dyspnea, fatigue, and confusion; he was found to have an oxygen saturation of 72% in the emergency department and subsequently placed on three liters of oxygen via nasal cannula. Initial chest x-rays in the emergency department demonstrated an enlarged heart and bilateral opacifications consistent with congestive heart failure (Figure 1). An electrocardiogram in the emergency department demonstrated normal sinus rhythm, left axis deviation, and a QTC of 483 milliseconds (Figure 2). A transthoracic echo performed on hospital day two demonstrated no evidence of valvular disease, a grade II diastolic dysfunction, and a left ventricular ejection fraction (LVEF) of 40-45%. Despite initial improvement, the patient became agitated and increasingly confused and was placed on average volume-assured pressure support. The arterial blood gas showed severe respiratory acidosis (Table 1), prompting the transfer of the patient to the critical care unit for further management. Upon transfer to the intensive care unit, further history was obtained from his spouse, who stated the patient had been using commercially available portable oxygen bottles to treat his shortness of breath symptoms. For multiple days before admission, he had been continuously using the supplemental commercially available portable oxygen bottles and had finished the whole bottle in a few days. During this time, the spouse noticed the patient developing worsening confusion and dyspnea.

**Table 1:** Arterial blood gases during admission PCO2: partial pressure of carbon dioxide; PO2: partial pressure of oxygen; HCO3: bicarbonate; FIO2: fraction of inspired oxygen; NC: nasal cannula

Hospital day:	pH	PCO2: normal range (35 - 48 mmHg)	PO2: normal range (83 - 108 mmHg)	HCO3: normal range (21 - 28 mmol/L)	Base excess: normal range (-2.0 - 3.0 mmol/L)	Oxygen saturation	FIO2
1	7.22	94 mmHg	138 mmHg	38 mmol/L	7.9 mmol/L	99.6%	50
2	7.31	81 mmHg	197 mmHg	40 mmol/L	11.4 mmol/L	197%	50
3	7.35	64 mmHg	65 mmHg	35 mmol/L	7.5 mmol/L	94.8%	28
4	7.38	60 mmHg	80 mmHg	36 mmol/L	8.6 mmol/L	96.8%	24 (2L NC)
5	7.59	40 mmHg	52 mmHg	38 mmol/L	15.2 mmol/L	24%	24
6	7.44	57 mmHg	83 mmHg	39 mmol/L	12.1 mmol/L	98.6%	33

The patient was treated in the intensive care unit for acute-on-chronic hypercapnic complications caused by carbon dioxide (CO2) narcosis, hypoxic respiratory failure, and heart failure exacerbations. The patient improved on non-invasive ventilation, and subsequent blood gases showed marked improvement (Table 1). Eventually, he was discharged home with a bilevel-positive airway pressure machine and referred to an outpatient pulmonologist for further management.

## Discussion

Commercially available portable oxygen bottles are advertised as supplemental oxygen, sold over the counter, and available at many major retail outlets. We believe that the excessive use of supplemental oxygen by this patient was the cause of his presentation to the emergency department (ED) with carbon dioxide narcosis.

Chronic obstructive pulmonary disease is when chronic inflammation of the airways, lung parenchyma, and pulmonary vessels results in tissue damage and structural changes to the airway and, over time, reduces lung function [3]. These chronic changes cause a ventilation-perfusion mismatch, which leads to hypoxemia and carbon dioxide retention [4].

Even though COPD is recognized as a common cause of hypercapnia caused by oxygen delivery, other disease processes can also make patients vulnerable to oxygen-induced hypercapnia [1,4]. The improper use of oxygen in patients with COPD exacerbations can be fatal, especially when high-flow oxygen concentrations are associated with increased mortality [5,6]. Many hypotheses have been studied that account for this phenomenon, but it is believed that ventilation-perfusion mismatch likely contributes the most and is potentially the driving cause of oxygen-induced hypercapnia [6]. Studies have shown that an increase in the oxygen concentration in the alveoli reduces vasoconstriction of the pulmonary vasculature, which increases ventilation-perfusion mismatch, which leads to alveolar dead space and a decrease in gaseous exchanging, hence responsible for oxygen-induced hypercapnia [6-8]. 

The Haldane effect is the result of oxygen causing an easier disassociation of carbon dioxide from hemoglobin, increasing the partial pressure of carbon dioxide in the blood. Typically, this can be compensated by increasing minute ventilation, but this is compromised in COPD patients, thereby increasing the carbon dioxide levels in the blood due to less excretion. The Haldane effect accounts for a small portion of hypercapnia caused by oxygen [6].

Hypercapnia in acute decompensated heart failure patients is associated with increased invasive airway intervention and increased hospital stay and mortality [9,10]. Patients with heart failure likely have reduced vital capacity due to fluid occupying air space, which leads to hypercapnia [11,12].

In our patient, we attribute it to the summation of the hypercapnic effects from his COPD, congestive heart failure (CHF), and non-prescribed supplemental oxygen use that resulted in dramatically rising CO2 levels.

## Conclusions

This case highlights the critical importance of cautious oxygen supplementation in patients with chronic obstructive pulmonary disease (COPD), mainly when using commercially available products without proper medical guidance. The patient's self-administration of commercially available portable oxygen bottles led to a cascade of events, resulting in hypercapnic respiratory failure and severe respiratory acidosis, necessitating intensive care. The authors suggest that high-risk patients, such as our presented patient, should only use supplemental oxygen with proper medical guidance and expert medical opinion. The primary care doctor is pivotal in ensuring proper management and guidance for patients with COPD and potential oxygen therapy needs. Primary care doctors are in a prime position to educate patients about the proper use of supplemental oxygen and the potential risks associated with its misuse. Patients with chronic respiratory conditions, such as COPD, may require oxygen therapy during their disease course. The patient's use of over-the-counter, commercially available portable oxygen bottles without medical supervision underscores the need for increased patient awareness regarding the potential dangers of self-administration. The primary care doctor can actively engage in patient education, emphasizing the importance of obtaining medical advice before initiating oxygen therapy. This includes discussing the appropriate devices for oxygen delivery, recommended flow rates, and potential adverse effects associated with oxygen use. The authors would also like to clarify that commercially available oxygen supplemental cans can prove beneficial in some circumstances, but medical opinion should be sought for high-risk patients.
